# A restricted signature of serum miRNAs distinguishes glioblastoma from lower grade gliomas

**DOI:** 10.1186/s13046-016-0393-0

**Published:** 2016-07-30

**Authors:** Giulia Regazzo, Irene Terrenato, Manuela Spagnuolo, Mariantonia Carosi, Gaetana Cognetti, Lucia Cicchillitti, Francesca Sperati, Veronica Villani, Carmine Carapella, Giulia Piaggio, Andrea Pelosi, Maria Giulia Rizzo

**Affiliations:** 1 Department of Research, Advanced Diagnostics and Technological Innovation, Genomic and Epigenetic Unit, Translational Research Area, Regina Elena National Cancer Institute, Via Elio Chianesi 53, 00144 Rome, Italy; 2 Biostatistical Unit, Regina Elena National Cancer Institute, Rome, Italy; 3 Department of Research, Advanced Diagnostics and Technological Innovation, Pathology Unit, Regina Elena National Cancer Institute, Rome, Italy; 4 Digital library, Knowledge Center “Riccardo Maceratini” and Patient Library, Regina Elena National Cancer Institute, Rome, Italy; 5 Department of Research, Advanced Diagnostics and Technological Innovation, SAFU Unit, Translational Research Area, Regina Elena National Cancer Institute, Rome, Italy; 6 Neuro-Oncology Unit, Regina Elena National Cancer Institute, Rome, Italy; 7 Department of Clinical and Experimental Oncology–Neurosurgery, Regina Elena National Cancer Institute, Rome, Italy

**Keywords:** Glioma, Biomarkers, Circulating microRNA, miR-497, miR-125b

## Abstract

**Background:**

Malignant gliomas are the most common primary brain tumors in adults and challenging cancers for diagnosis and treatment. They remain a disease for which non-invasive, diagnostic and/or prognostic novel biomarkers are highly desirable. Altered microRNA (miRNA) profiles have been observed in tumor tissues and biological fluids. To date only a small set of circulating/serum miRNA is found to be differentially expressed in brain tumors compared to normal controls.

Here a restricted signature of circulating/serum miRNA including miR-15b*,-23a, −99a, −125b, −133a, −150*, −197, −340, −497, −548b-5p and let-7c were investigated as potential non-invasive biomarkers in the diagnosis of glioma patients.

**Methods:**

Serum and tissues miRNAs expression in patients with brain cancers (*n =* 30) and healthy controls (*n =* 15) were detected by quantitative real-time polymerase chain reaction (qRT-PCR). Relative expression was calculated using the comparative Ct method. Statistical significance (*p* ≤ 0,05) was determined using the Mann–Whitney rank sum and Fisher’s exact test. Diagnostic accuracy of miRNAs in distinguishing glioblastoma multiforme (GBM) from lower grade cancer was assessed by the Receiver Operating Characteristic (ROC) curve analysis.

To validate the role of the identified miRNAs in cancer a comprehensive literature search was conducted using PubMed, Web of Science (Core Collection) and Scopus databases.

**Results:**

We observed a decrease of miR-497 and miR-125b serum levels depending on tumor stages with reduced level in GBM than lower grade tumors. The ROC curve analysis distinguishing GBM from lower grade cases yielded an area under the curve (AUC) of 0.87 (95 % confidence interval (CI) = 0.712–1) and of 0.75 (95 % CI = 0.533–0.967) for miR-497 and -125b, respectively. GBM patients are more likely to show a miR-497 and -125b down-regulation than the lower grade group (*p =* 0.002 and *p =* 0.024, respectively). These results were subsequently compared with evidence from 19 studies included in the final systematic review.

**Conclusions:**

Although multiple biomarkers are currently leveraged in the clinic to detect specific cancer types, no such standard blood biomolecules are used as yet in gliomas. Our data suggest that serum miR-497 and -125b could be a novel diagnostic markers with good perspectives for future clinical applications in patients with glioma.

## Background

Malignant gliomas and mainly glioblastomas multiforme (GBMs), represent the most lethal primary brain tumors and are associated with high morbidity, presenting unique challenges to therapy due to their location, aggressive biological behaviour and diffuse infiltrative growth [1, 2] according to current World Health Organization (WHO) guidelines, gliomas are subcategorized by histopathologic evaluation into four tumor grades (I–IV), where GBM (grade IV) is the most malignant and most common primary brain tumor [3]. Currently, histological examination of the tumor tissue is the standard procedure for definitive diagnosis of glioma, while computer tomography and nuclear magnetic resonance imaging (MRI) are the supplement procedures for disease staging [4–6]. However, both the histopathology and neuroimaging tests are insensitive and expensive, and may cause haemorrhage and neurologic damages [7]. Thus, there is a major interest in developing biomarkers that allow for less expensive monitoring of the disease in shorter time intervals [8]. Furthermore, patients at high risk for surgery-associated mortality or small tumors in eloquent areas of the brain could benefit from the discovery of biomarkers for the confirmation of GBM in order to avoid biopsy [9]*.* Although early detection with circulating biomarkers is an established method in the diagnosis and treatment of many cancer types, diagnostic tests for gliomas are currently limited [10]. Therefore, to have a set of markers for early detection is still a primary goal to improve diagnosis and treatment of human gliomas.

MicroRNAs (miRNAs) are small non-coding RNAs that play an important role in the regulation of various biological processes through their interaction with cellular messenger RNAs [11]. They are frequently deregulated in cancer [12–14] and have shown great potential as tissue-based markers for cancer classification and prognosis including gliomas [15–17]. MiRNAs are also present in extracellular human body fluids such as blood serum and plasma [18, 19]. Since miRNAs circulate in the bloodstream in a highly stable, extracellular form, they have become particularly appealing as potential non-invasive biomarkers for detection of cancer [20–22].

Regarding gliomas, plasma miR-21, one of the most intensively studied miRNAs in cancer biology, has been found significantly higher in GBM patients than control samples and it decreased significantly after tumor resection and concomitant radio/chemotherapy [23–25]. Recently, a global serum miRNA signature in a large cohort of malignant glioma patients has been reported. In particular, seven serum miRNAs (miR-15b*, miR-23a, miR-133a, miR-150*, miR-197, miR-497 and miR-548b-5p) whose concentrations were significantly decreased in serum of malignant astrocytoma patients compared to normal controls have been identified [26]. Moreover, differentially expressed serum miRNAs were found in GBM patients and normal controls. In particular, miR-576-5p, miR-340 and miR-626 were significantly overexpressed, whereas miR-320, let-7 g-5p and miR-7-5p showed significantly low expression in GBM patients [27]. Besides, Wu J *et al.* reported a predictive value of serum miR-29 in a high-graded glioma screening [28]. Lastly, miR-125b, a member of the let-7c cluster widely considered as ideal biomarker for clinical diagnosis of various human cancers [29–31], has also been described as potential glioma biomarker [32].

Although all these promising circulating biomarkers have been suggested to have a prognostic and diagnostic value for glioma tumors, they should be further tested and evaluated in order to be widely accepted.

Here, we assessed the suitability of eleven selected serum miRNAs, previously shown to be associated with brain tumors [26, 27, 32], as potential non-invasive diagnostic tools for glioma patients.

## Methods

### Study design, patients and control subjects

The present study involved two phases. In the first phase, the predictive performance of a restricted signature of serum miRNAs (miR-15b*, −23a, let-7c, −99a, −125b, −133a, −150*, −197, −340, −497 and miR-548b-5p) for glioma was assessed.

Preoperative serum samples were collected from 30 newly diagnosed brain tumors (22 primary glioma patients of WHO grades II-IV, 8 meningiomas) at the Regina Elena National Cancer Institute. Healthy controls (*n =* 15) were recruited at the same Institute from individuals seeking a routine health checkup and with no evidence of disease and with age-, gender- and ethnicity-matched to the patients.

Formalin-fixed and paraffin-embedded (FFPE) human non-neoplastic brain tissue from autopsies and glioma tissue matched to serum of patient included in the study, were obtained from the Regina Elena National Cancer Institute. The clinic-pathological features of all the patients are summarized in Table 1.

**Table 1:** General characteristics and clinicopathological features of brain tumor patients and control subjects

Variables		Controls(*n =* 15)	Gliomas(*n =* 22)	Meningiomas (*n =* 8)
Age	mean	51	54	61
	range	29–65	24–82	27–76
Gender	males	8	12	3
	females	7	10	5
Neurological disorders^a^		_	6	_
Grade	II/III	_	12	_
	IV	_	10	_
IDH1 mutation^b c^		_	6	_
IDH2 mutation^c^		_	0	_
MGMT promoter methylated^d^		_	12	_

^a^ Hemisyndrome, psychiatric disorders, cognitive disorders

^b^ mutation r132h

^c^ 19 out of 22 patients analyzed for this mutations ^d^ MGMT promoter was considered methylated if the percentage of methylation was >5 %

14 out of 22 patients analyzed for methylation

All participants gave written informed consent and the Research Ethics Committee of Regina Elena National Cancer Institute approved this investigation. Patient data and samples were treated according to ethical and legal standards adopted by the Declaration of Helsinki 2013.

In the second phase of the study an on-line literature search, for miR-497 and miR-125b, was conducted in PubMed (from 01/01/1946 to 05/01/2016), Web of Science (Core Collection) (from 01/01/1990 to 05/01/2016) and Scopus (from 01/01/2004 to 05/01/2016) databases to obtain all the studies related to the predictive diagnostic performance of these miRNAs in different cancers. Our search strategy was based on a combination of keywords and Boolean operators organized as follows: **(1)** MicroRNA* OR “Micro RNA” OR “RNA Micro” OR miRNA* OR pri-miRNA* OR stRNA* OR pre-miRNA*; **(2)** Small AND Temporal AND RNA*; **(3)1** OR **2; (4)** 125b; **(5)3** AND **4; (6)** miR-125b OR microRNA-125b OR hsa-mir-125b OR miRNA-125b OR micro-RNA-125b OR pri-miRNA-125b OR stRNA-125b OR pre-miRNA-125b; **(7)5** OR **6; (8)** “MicroRNAs”[Mesh]) AND 125b; **(9)7** OR **8; (10)**neoplas* OR tumo* OR cancer*; **(11) 9** AND **10**.

In Scopus and Web of Science MeSH terms were not included in the search. The same strategy has been replicated for miR-497 without passage 10 and 11 because, considering the smaller number of citations found for this miRNA, it was not necessary to further restrict the search including the two final passages of the search strategy.

Three review authors independently screened titles and abstracts in order to identify articles for eligibility. Later the review authors independently screened the full-text articles for eligibility. Both the screening for titles and abstracts and the full-text screening were performed using a standardized form with explicit inclusion and exclusion criteria. Studies from the initial literature search have been considered qualified and included in our review if they satisfied the following criteria: (1) full articles in English (reviews were excluded); (2) articles concerning the evaluation of cell-free miRNA in human biofluids from cancer patients; (3) recruitment of more than 10 participants for the study (4) sample collection before surgical resection or other therapeutic treatments; (5) no declared concomitance with other diseases; (6) analysis of miRNA levels for diagnostic purposes. The review authors resolved any disagreement by discussion.

### Sample processing and RNA extraction

Blood samples of glioma patients were collected before surgery, radiotherapy or chemotherapy in BD Vacutainer serum tubes using a 21-gauge needle. The samples were kept at room temperature (RT) for 30–60 min and then centrifuged at RT for 20 min at 1200 g. The serum transferred into sterile cryovials was aliquoted and stored at −80 °C until further analysis.

To isolate extracellular circulating miRNA in serum we used a kit that permits to obtain all circulating cell-free miRNAs both from exosomes and associated proteins (Ago proteins, nucleoplasmin, HDL, etc.). RNA was extracted from 200 μl of serum and purified using miRCURY RNA Isolation Kit – Biofluids (Exiqon #300112 Vedbaek- Denmark) in accordance with manufacturer’s instructions. There is considerable sample-to-sample variability in both protein and lipid content of plasma and serum samples, which could affect efficiency of RNA extraction, and could introduce potential inhibitors of PCR. In order to minimize the technical variation between replicates in down-stream PCR analysis we added, for all isolations, spike-in non-human synthetic miRNAs (RNA spike-in mix: UniSp2, UniSp4 and UniSp5; Exiqon #203203) into the respective lysis/denaturant buffer before combining with serum. To avoid DNA contamination, all samples were subjected to on-column rDNase treatment in accordance with manufacturer’s instructions. After extraction RNA was eluted in 50 μl RNase-free water. Furthermore, determination of RNA yield is usually not possible by spectrophotometric reading thus we used RNA amounts based on starting volume in the PCR reaction as a measure, combined with subsequent quantification of spike-ins. RNA from FFPE samples were extracted using the PureLink FFPE Total RNA Isolation Kit (Invitrogen. Carlsbad, CA, USA) in accordance with manufacturer’s instructions. Total tissue RNA, eluted in RNase-free water, was quantified with the NanoDrop ND-1000 spectrophotometer (ThermoFisher Scientific, Wilmington, DE U.S.A.).

### Reverse transcription and quantitative real-time -PCR (qRT-PCR)

Quantification of the mature circulating and tissue miRNAs were performed by a miRNA-specific LNA™-based system using SYBR® Green (miRCURY LNA™ Universal RT microRNA PCR; Exiqon # 203301, 203351 Vedbaek- Denmark).

First-strand cDNA was synthesized from 4 μl of each serum RNA sample or 20 ng of tissue RNA, using the Universal cDNA Synthesis kit II according to the Exiqon manufacturer’s protocols with any modifications (Exiqon, Vedbaek- Denmark). To control the potential presence of inhibitors and the quality of the cDNA synthesis reaction UniSp6 RNA Spike-in template was added to the Reverse Transcription mixture.

The cDNA template was diluited 40x in nuclease-free water and then amplified using microRNA-specific LNA™-enhanced forward and reverse primers. QRT-PCR was performed employing an ABI 7900 Real Time PCR System and SDS 2.2.2 software (Applied Biosystems, Foster City, CA). All reactions were performed in triplicate and for the background level a No Template Control was included in the study every time a new experiment was set-up. ROX passive reference dye was added in the diluited cDNA samples to obtain a robust read over the entire array of wells (ROX high, Kapabiosystems, Wilmington, Massacchusetts, USA). Expression data for miRNAs were analyzed calculating cycle threshold (Ct) values as well as standard deviations by means of comparative ΔCt method. The quantity of serum and tissue miRNAs was normalized as described below.

Established consensus house-keeping miRNAs for data normalization are lacking for serum miRNAs. Exiqon manufacturer’s protocols suggest miR-103-3p as a candidate endogenous reference gene but it showed high variation in our samples. Thus, in order to minimize variation in circulating miRNA recovery, retro-transcription and amplification efficiency, we normalized serum miRNA levels measuring the expression of the synthetic spiked-inUniSP2 (UniSP2 LNA control primer set UniRT, Exiqon#203950).

The expression levels of mature tissue miRNAs were normalized to the U6 snRNA (U6 snRNA LNA primer set UniRTExiqon #203907).

As the major source of variation in plasma and serum miRNA expression patterns is potential cellular derived miRNA contamination including hemolysis [33] we screened selected samples for hemolysis analyzing the expression levels of miR-451, abundant in red cells, and miR-23a, unaffected by hemolysis, as suggested by Exiqon manufacturer’s protocols (samples with Ct miR-23a – Ct miR-451 ≥ 5 are considered hemolyzed).

### Statistical analyses

Statistical significance (*p* ≤ 0,05) was determined using the Mann–Whitney rank sum and Fisher’s exact test, when appropriate. MiRNA expression values were plotted into box-plots. Receiver operative characteristics (ROC) curves were used to assess the diagnostic accuracy of miRNAs in distinguish high grade from lower grade cancer. Youden’s index was performed to identify the best cut-off value. Across various cut-off points, Youden’s index maximized the difference between sensitivity and specificity and between real-positive and false-positive subjects. All analyses were carried out with SPSS (version 21.0) statistical program (SPSS Inc., Chicago, IL, USA).

## Results and Discussion

The conventional method for glioma detection is neuroimaging, which is considered as gold standard but can hardly avoid limitations such as invasive process, expensive cost, and unwarrantable accuracy [5, 6, 21, 34]. Therefore, to have a set of markers for early detection is still a primary goal to improve diagnosis and treatment of human gliomas. To overcome these shortcomings, biomarkers in blood have become a hot research field. Since most current protein biomarkers cannot reach certain level of sensitivities or specificities, miRNAs, which can be sampled non-invasively and cost-effectively, are widely studied as ideal biomarkers for clinical applications [35].

### Expression level of a selected subset of serum miRNAs in a cohort of glioma patients

Circulating miRNAs, previously shown to be associated with brain tumors, were evaluated as potential non-invasive diagnostic biomarkers in the serum of a cohortofpatients with brain tumors including gliomas of different grade (*n =* 22), meningiomas (*n =* 8) and normal control subjects (*n =* 15) with a matched distribution of age and sex. An overview of the patient characteristics is provided in Table 1.

**Fig. 1:**
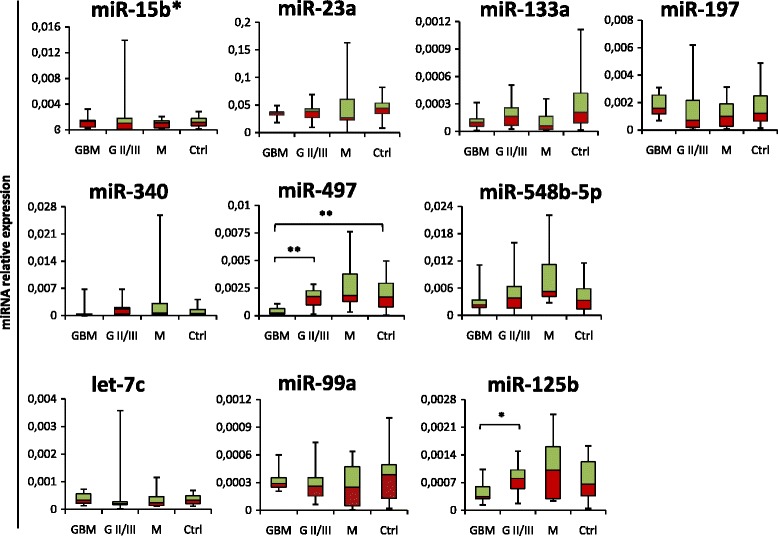
Expression levels of selected miRNAs in a cohort of patients with brain tumors. Box-plot diagrams of relative miRNA expression levels in pre-operative serum samples from glioblastoma (GBM; *n =* 10), lower grade glioma (G II/III; *n =* 12), meningioma (M; *n =* 8) patients and healthy subjects (Ctrl; *n =* 15), assessed by qRT–PCR. Boxes define the 25th and 75th percentiles; the horizontal line into the boxes indicates the median, and bars define the minimum and maximum values. The expression levels of mature miRNAs were normalized to volume and UniSp2 spike-in RNA. Relative expression was calculated using the comparative ΔCt method. *p*-values (* = *p* ≤ 0,05; ** = *p* ≤ 0,01) were determined using the Mann–Whitney rank sum test

In particular, we profiled by qRT-PCR the expression level of eight serum miRNAs selected from the literature (miR-15b*, −23a, −133a, −150*, −197, −340, −497 and miR-548b-5p [26, 27] and three miRNAs from empirical data from in-house studies (let-7c, −99a, −125b). As shown in Fig. 1, we found that in GBM patients only serum miR-497 was significantly down-regulated (−4,8 folds; *p* ≤ 0.01) compared to healthy donors. Interestingly, when patient samples were clustered based on histological grade, a statistically significant decrease of the mean serum miR-497 expression (−3,75 fold; 73.3 %) but also of miR-125b (−1,8 fold; 43.6 %) was evident in high-grade relative to lower grade samples. Moreover, benign brain tumors as meningiomas showed levels of these miRNAs comparable to control samples (Fig. 1). IDH mutation and MGMT methylation are grade-dependent markers in glioma specimens [36, 37]. Interestingly, regarding IDH1 mutation status, we observed that patients who carry the IDH1 R132H mutation, usually associated with a lower grade glioma [38], are more likely to have levels of miR-125b and −497 higher while patients carrying IDH-wild type gene have levels of miR-125b and −497 lower than the cut-off values (data not shown). As far as methylation status, it is of interest to note that, although lacking strong statistical significance due to the low number of samples available, the unmethylated condition, that is described to be associated with a resistence to chemotherapy [38], seems to be tendentially associated with levels of miR-125b and −49**7** lower than the cut-off values (data not shown).

These results suggest a possible diagnostic application of miR-497 and -125b in determining the tumor grade in gliomas before biopsy or surgical resection, that are usually required for histopathological classification and grading [10].

However, our analysis did not recapitulate, except for miR-497 and miR-125b, the results of previous studies [26, 27] that identified all the miRNAs of the signature as modulated in gliomas compared with normal control. This contrastating data may be explained with the several challenges in studying circulating miRNA. In fact, while we are confident that in the case of miR-340 the main key limiting factor for the absence of modulation in glioma is the small sample size of the Dong et al. study (miRNA profiles from only 3 circulating blood samples of GBM patients and 3 matched healthy controls) for the other selected miRNAs the conflicting results may resides on the many factors that can affect the analysis and the evaluation of circulating miRNAs expression [25, 39].

A major obstacle to the translation of circulating miRNAs from laboratory studies into the clinic is the lack of consistent and robust results with many apparently contradictory reports in the literature [40]. A likely reason for this lack of reproducibility is that there are very few multi-center studies, and cohorts are often insufficiently powered. Another confounding factor is the fact that there is a high degree of inter-individual variability in the levels of circulating miRNAs, even when focusing only on healthy populations [41]. Moreover, there is a technical source of variation between studies, such as the starting material used for the experiments (e.g., the choice of use serum or plasma, the RNA extraction method, the normalization, etc.), the technological platforms [e.g., microarray, qRT-PCR vs. next generation sequencing (NGS) etc.], and the differing statistical methodologies used.

### Diagnostic accuracy of serum miR-497 and miR-125b

**Fig. 2:**
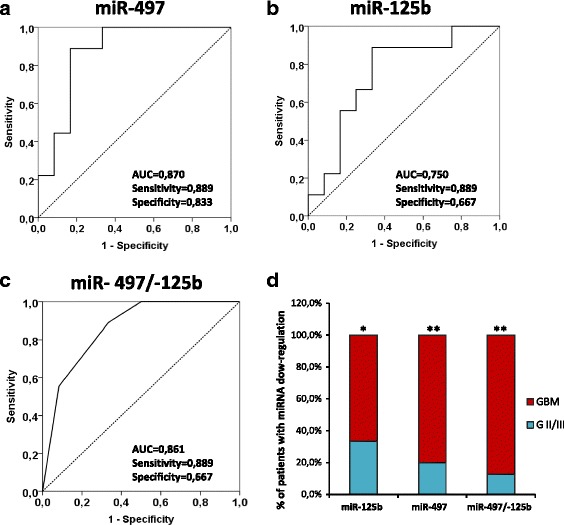
ROC curves of serum miR-125b and miR-497 to discriminate glioblastomas from lower grade gliomas. **a-b** ROC curve plotted for diagnostic potential and discriminatory accuracy of serum miR-497 and miR-125b to distinguish GBM from G II/III gliomas. The corresponding AUC, sensitivity and specificity values are reported. **c** ROC curve for the combined miR-497/-125b panel by a dummy variable with value 1 for patients with decrement of both miRNAs and 0 for the other combinations. **d** The histogram represents the distribution, between GBM and lower grade gliomas (G II/III), of the patients with miRNA expression levels lower than the cut-off values (0,00083 for miR-497 and 0,00065 for miR-125b). *p*-values (* = *p* ≤ 0,05; ** = ≤ 0,01) were determined using the Fisher’s exact test

To further explore the diagnostic potential and discriminatory accuracy of serum miR-497 and miR-125b in glioma patients, the ROC curves were established. The ROC analyses revealed that serum miR-497 and miR-125b levels were robust in discriminating patients with GBM from lower grade gliomas, with an AUC value 0.87 (95 % CI = 0.712– 1) and of 0.75 (95 % CI = 0.533–0.967) respectively (Fig. 2 a, b). The highest accuracy was at a cut-off expression value 0.00083 (miR-497) and 0.00065 (miR-125b), where the negative predictive value, positive predictive value, sensitivity, and specificity to identify a patient with GBM were 0.909, 0.800, 0.889, 0.833 for miR-497 and 0.889, 0.667, 0.889, 0.667 for miR-125b. We became interested in evaluating if the combination of miR-497 and miR-125b could have an increased diagnostic accuracy. For this purpose, a miRNA panel was created by a dummy variable assigning value 1 to the patients who had a decrement of both the miR-497 and -125b and 0 for the other combinations (Fig. 2c).

The cut-off used to identify patients with modulated miRNAs were obtained from the ROC curves shown in Fig. 2a and b. Our analysis indicate that no substantial improvement in the diagnostic performance was observed between single or combined use of the two miRNAs (Fig. 2c). This is mainly due to the excellent performance of miR-497 (AUC = 0.870) that flattens the result of miR-125b (AUC = 0.750) which however remain a good marker. Nevertheless, taking into account the patients with an expression level of miRNAs lower than the cut-off value, the number of subjects belonging to the group of GBMs is significantly higher than the number of patients affected from glioma GII/III (Fig. 2d). This difference can be observed for both miR-497 and -125b and is more evident if we take into consideration the patients with both miRNAs down-regulated.

**Fig. 3:**
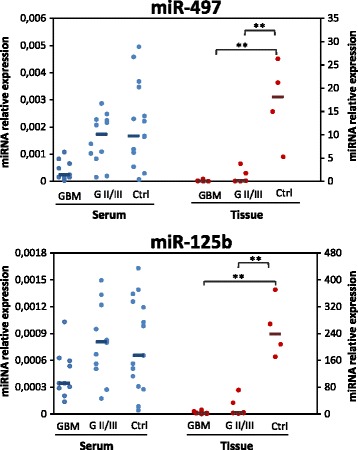
Expression levels of miR-497 and miR-125b in serum and matched tissue samples of glioma patients. Scatter plots of the normalized expression levels of miR-497 and -125b. The left ordinate axis: miRNA levels in serum; the right ordinate axis: miRNA levels in tissue samples. The horizontal line indicates the median. A Mann–Whitney *U* test was conducted to compare the levels of miRNAs in tumor samples (GBM *n =* 9; GII/III *n =* 6) and normal brain tissues (Ctrl *n =* 4). Mature miRNAs were normalized to volume and UniSp2 spike-in RNA for serum and to U6 snRNA for tissue miRNAs. Relative expression was calculated using the comparative ΔCT method. *p*-values (** = *p* ≤ 0,01) were determined using the Mann–Whitney rank sum test

As the comparison of the miRNA expression patterns between serum and tissues may provide additional evidence supporting the use of serum miRNAs as reliable diagnostic biomarkers, we examined the expression of miR-497 and -125b in paired tissue samples of glioma patients and in normal brain from autopsy. Consistent with the results in serum, we found decreased expression of the two identified miRNAs in tumor samples relative to normal, post-mortem brain tissues (Fig. 3). Thus, these data can indirectly support the advantage of our miRNA detection technique in serum compared to those used in previously published reports [26, 27]. However, while miR-497 and miR-125b levels in serum distinguish high-grade from lower grade glioma, in tumor samples they only distinguish tumor from normal samples. This different results may reflect a yet unknown biological phenomenon of physiological significance ranging from the nature of the blood as a systemic biofluid to the relative amount/stability that miRNAs can have in different body districts [42]. Moreover the detailed knowledge of the molecular mechanisms governing miRNAs release from normal and tumour tissues is still lacking since the debate about the relative contribution of organs and/or tissue to miRNAs in blood plasma is still open [18, 40, 43]. Anyway, abundant evidence shows that circulating nucleic acids in oncologic patients are dramatically altered with respect to healthy individuals, suggesting that the tumour bulk could be the major contributor in nucleic acid release in bloodstream [44, 45]. In addition, we can hypothesize that the tumor mass could indirectly affect circulating miRNA profiles inducing or inhibiting non neoplastic tissues to release biomolecules in blood.

### Systematic review of the diagnostic value of miR-497 and miR-125b in tumors

**Fig. 4:**
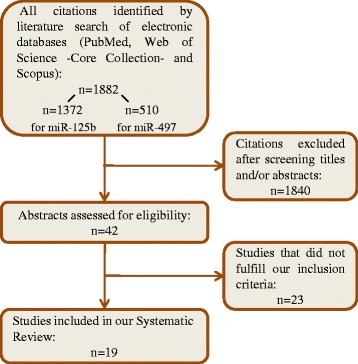
Flow diagram of the study selection process

Finally, a systematic review was conducted in order to have an overview on the role of the two identified miRNAs in cancer and to validate their possible use as non-invasive biomarkers for diagnostic purposes. For this reason we performed a literature research in multiple databases with the aim of evaluating the results of all the available articles concerning the expression of miR-497 and -125b in human cancers, focusing on the studies conducted only in biofluid samples. After having performed the screening of titles and abstracts of all the citations obtained by the initial literature search (Fig. 4), a total of 42 full-text articles were retrieved and 19 of them fulfilled the inclusion criteria described in the “Methods” section and were eligible studies for the systematic review. All the articles included in the final review are reported in Table 2 where the main characteristics and the results of each study are also summarized in terms of tumor or biofluid type and comparison of the level of miRNA between cases and controls [26, 32, 46–62].

**Table 2:** Studies included in the systematic review

MicroRNA	Cancer type	Biofluid type	Deregulation	Author	Reference
miR-125b	Bladder cancer	Urine	↓ tumors *vs* controls	Zhang *et al*.	46
			↓ high-grades *vs* low-grades		
	Uveal Melanoma	Plasma	↑tumors *vs* controls	Achberger *et al.*	47
	Advanced Melanoma	Serum^a^	↓tumors *vs* controls	Alegre *et al.*	48
	Ewing’s sarcoma	Serum	↓ tumors *vs* controls	Nie *et al.*	49
	Breast cancer	Serum	↑ late stages vs controls	Wang *et al.*	50
			↑ late stages *vs* early stages		
	Breast cancer	Urine	↓ tumors *vs* controls	Erbes *et al.*	51
	Breast cancer	Serum	↑ tumors *vs* controls	Mar-Aguilar *et al.*	52
	Breast cancer	Serum	↑ tumors *vs* controls	Matamala *et al.*	53
	Non-small-cell lung cancer	Serum	↑ tumors*vs* controls	Yuxia *et al.*	54
			↑ late stages *vs* early stages		
	Non-small-cell lung cancer	Plasma	↑ tumors *vs* controls	Zhao *et al.*	55
			↑ late stages *vs* early stages		
	Diffuse large B-cell lymphoma	Serum	↑ tumors *vs* controls	Yuan *et al.*	56
	Laryngeal squamous cell carcinoma	Plasma	↓ tumors *vs* controls	Ayaz *et al.*	57
	Glioma	Serum	↓ tumors *vs* controls	Wei *et al.*	32
			↓ high-grades *vs* low-grades		
	Colorectal neoplasia	Serum	↑ tumors *vs* controls	Yamada *et al.*	58
miR-497	Bladder cancer	Plasma	↓ tumors *vs* controls	Du *et al.*	59
	Astrocytoma	Serum	↓ tumors *vs* controls	Yang *et al.*	26
	Nasopharyngeal carcinoma	Plasma	↓ tumors *vs* controls	Wang *et al.*	60
	Prostate cancer	Serum	↓ tumors *vs* controls	Kong *et al.*	61
	Cervical cancer	Serum	↑ tumors *vs* controls	Zhang *et al*.	62

^**a**^Exosomal fraction only

MiR-497 is organized in a cluster with miR-195, located on chromosome 17p13.1 [63]. It belongs to the miR-15/107 family whose members share the same seed sequence end are widely expressed in many human tissues [64] and in particular in brain tissues miR-497 has been reported expressed and functionally relevant in mouse [65]. Its role in cancer has been investigated in many tumor tissues and cell lines [63, 66–68]. In the majority of these studies miR-497 is reported to have a tumor suppressor activity and regulates numerous pathways involved in cellular proliferation, migration, invasion and in angiogenesis [60, 66, 67] For example this miRNA is dowregulated in bladder cancer where it represses the inhibitor of apoptosis BIRC5 [63]. Moreover it inhibits angiogenesis and metastasis formation in a murin model of hepatocellular carcinoma through direct repression of VEGFA and AEG-1 protein level [66]. So far very few studies have been conducted about the role of miR-497 in gliomas. Lan *et al.* reported it overexpressed in glioma tissues and cell lines in response to hypoxia and observed a relationship between miR-497 upregulation and resistance to temozolomide [69]. On the other hand this miRNA, thanks to its repressive action on VEGFA, seems to mediate the inhibition of glioblastoma tissue vascularisation determined by ginsenoide Rh2 in an *in vivo* murine model [70]. According to our systematic review, miR-497 expression in biofluids appears to be so far less investigated than miR-125b, the other miRNA evaluated in the present study. All the studies shown in Table 2 are conducted on blood samples and the majority of them report miR-497 to be downregulated in patients compared to healthy controls [26, 59–61]. In the experiments performed by Yang *et al*. in astrocytoma patients miR-497 appears to be underexpressed in GBM serum samples in accordance with our results [26].

Regarding miR-125b, naturally express at high levels in brain tissue and in the ovarian tissue [71], it is highly conserved among mammals, vertebrates and nematodes [72]. In humans, as in most of the genomes, there are two paralogs (hsa-miR-125b-1 on chromosome 11 and hsa-miR-125b-2 on chromosome 21), coding for the same mature sequence. MiR-125b-2 is organized in a cluster with miR-99a and let-7c [73].

MiR-125b has been observed to be deregulated in solid tumors, for example it is overexpressed in prostate [74], colorectal cancers [75] and non-small-cell lung cancers [76], whereas it is downregulated in breast [77], oral cancers [78], melanoma [79], hepatocellular [80], thyroid anaplastic carcinomas [81], and bladder cancer [82]. The role of miR-125b in gliomas appears to be controversial. While it is described as an oncogene [83, 84] it is reported also to be downregulated in glioma stem cells and to have an anti-angiogenic role [85]. This controversial role is described also in the studies about miR-125b expression in biofluids [32, 46–58]. In accordance with our data, experiments conducted in glioma from Wei *et al.* report miR-125b to be downregulated in serum tumor samples [32]. Surprisingly, in our cohort of subjects, the expression of miR-125b in not correlated with that of the other members of the cluster, miR-99a and let-7c. This could be explained by the fact that the same mature form of miR-125b is encoded not only in the let-7c cluster but also in another locus on chromosome 11. Interestingly, in the context of bliofluids, the expression of miR-125b seems to be dependent not only from the type of tumor but also from the kind of biofluid examined: as an example in three out of four studies concerning breast cancer, conducted on blood derived biofluids, miR-125b is reported to be upregulated in serum tumor samples [50, 52, 53], while it is described as downregulated in the only study conducted on urine [51]. This dependence of miRNA expression on the biofluid type has already been described for other miRNAs [25]. Moreover, miR-125b seems to have a possible dual role depending on the type or cellular context: it can act as both an oncomiR or a tumor suppressor miR by targeting tumor suppressor genes or oncogenes respectively. For example, on the one hand miR-125b targets multiple genes involved in the p53 pathway and induces a blockage of apoptosis in human neuroblastoma cells [29, 71, 86], on the other hand it can negatively affects the expression of proteins involved in the regulation of cellular proliferation as E2F3, ERBB2/3 [87] or the anti-apoptotic protein Bcl2 [71], suggesting an oncosuppressive role. Thus, the results of our systematic review join a current trend to include circulating miR-497 and miR-125b as good non-invasive diagnostic biomarkers for several tumors [32, 47, 60]as they appear to be deregulated in association with a variety of cancer types. This is valid also in the case of miR-125b whose direction of deregulation could be different in different kinds of tumor, due to the dual role of this miRNA as oncomiR or tumor suppressor depending on cellular type or context [71, 76].

## Conclusions

Although multiple biomarkers are currently leveraged in the clinic to detect specific cancer types, no such standard blood biomolecules have been identified in glioma patients [9, 10]. Thus, it is clear that identification of new molecular glioma biomarkers with the aim of developing minimally invasive tests for the detection and monitoring of glioma patients are demanded.

In this study we underlined the role of serum miR-497 and -125b as diagnostic markers and as mirrors of tissue miRNA level in gliomas thus suggesting the clinical potential of these miRNAs to discriminate with high sensitivity and specificity the GBM from lower grade gliomas. Nevertheless, challenges remain before miR-497 and -125b are leveraged as bona fide biomarkers in glioma cancers and further investigation from larger independent studies is needed to unveil their clinical relevance.

## Abbreviations

AUC, area under the curve; CI, confidence interval; Ct, cycle threshold; FFPE, Formalin-fixation and paraffin-embedding; GBM, glioblastoma multiforme; miRNA, microRNA; MRI, nuclear magnetic resonance imaging; NGS, next generation sequencing; qRT-PCR, quantitative real-time polymerase chain reaction; ROC, receiver operating characteristic; RT, room temperature; WHO, World health organization
